# Local exposure misclassification in national models: relationships with urban infrastructure and demographics

**DOI:** 10.1038/s41370-023-00624-z

**Published:** 2023-12-22

**Authors:** Sarah E. Chambliss, Mark Joseph Campmier, Michelle Audirac, Joshua S. Apte, Corwin M. Zigler

**Affiliations:** 1 Department of Statistics and Data Sciences, The University of Texas at Austin, Austin, TX 78712 USA; 2 Department of Civil and Environmental Engineering, University of California, Berkeley, Berkeley, CA 94720 USA; 3 Department of Biostatistics, Harvard T.H. Chan School of Public Health, Boston, MA 02115 USA; 4 School of Public Health, University of California, Berkeley, Berkeley, CA 94720 USA

**Keywords:** Air pollution, Environmental justice, Analytical methods, Exposure modeling, Geospatial analyses, Particulate matter

## Abstract

**Background:**

National-scale linear regression-based modeling may mischaracterize localized patterns, including hyperlocal peaks and neighborhood- to regional-scale gradients. For studies focused on within-city differences, this mischaracterization poses a risk of exposure misclassification, affecting epidemiological and environmental justice conclusions.

**Objective:**

Characterize the difference between intraurban pollution patterns predicted by national-scale land use regression modeling and observation-based estimates within a localized domain and examine the relationship between that difference and urban infrastructure and demographics.

**Methods:**

We compare highly resolved (0.01 km^2^) observations of NO_2_ mixing ratio and ultrafine particle (UFP) count obtained via mobile monitoring with national model predictions in thirteen neighborhoods in the San Francisco Bay Area. Grid cell-level divergence between modeled and observed concentrations is termed “localized difference.” We use a flexible machine learning modeling technique, Bayesian Additive Regression Trees, to investigate potentially nonlinear relationships between discrepancy between localized difference and known local emission sources as well as census block group racial/ethnic composition.

**Results:**

We find that observed local pollution extremes are not represented by land use regression predictions and that observed UFP count significantly exceeds regression predictions. Machine learning models show significant nonlinear relationships among localized differences between predictions and observations and the density of several types of pollution-related infrastructure (roadways, commercial and industrial operations). In addition, localized difference was greater in areas with higher population density and a lower share of white non-Hispanic residents, indicating that exposure misclassification by national models differs among subpopulations.

**Impact:**

Comparing national-scale pollution predictions with hyperlocal observations in the San Francisco Bay Area, we find greater discrepancies near major roadways and food service locations and systematic underestimation of concentrations in neighborhoods with a lower share of non-Hispanic white residents. These findings carry implications for using national-scale models in intraurban epidemiological and environmental justice applications and establish the potential utility of supplementing large-scale estimates with publicly available urban infrastructure and pollution source information.

## Introduction

Better representation of within-city air pollution gradients—necessary to reduce exposure misclassification in epidemiological studies [1–4] and better quantify exposure disparity in environmental justice research [5–9]—has motivated major innovation in both modeling and measurement methods [10–17]. Currently available modeled products for the United States offer both broad geographic coverage and fine scale intraurban spatial resolution [17–19]. In theory, this facilitates an assessment of exposure variation within any city in the continental US. However, when these high-resolution products are used to estimate exposure within a single urban area or other model subdomain, they may confer patterns of exposure misclassification that differ from those evaluated over a larger geographic domain. For studies focused on characterizing within-city exposure differences, it is important to examine whether national-scale predictions are more susceptible to misestimation near certain pollution-related infrastructure and whether that misestimation differentially affects exposure estimates among subpopulations.

Challenges in characterizing intraurban gradients vary among pollutants. For PM_2.5_ estimates, the application of modeling and remote sensing—including satellite-based remote sensing [14], reduced complexity mechanistic models [16], and ensemble machine learning techniques [17]—has generally provided moderate intraurban resolution (1 km^2^). Because of the strong secondary contribution to PM_2.5_, variation at the <1 km scale is relatively low and this spatial resolution adequately characterizes intraurban gradients [20]. In contrast, the small-scale atmospheric dynamics of a reactive pollutant with strong near-source peaks, such as oxides of nitrogen (NO and NO_2_) and ultrafine particle count (UFP), can result in two- to ten-fold decay at a distance of 50 m to 300 m from a source such as a highway [21, 22]. The markedly different short-scale spatial patterns of NO_2_ and UFP compared with urban PM_2.5_ carry implications for both exposure modeling and health. NO_2_ may harm the body along a different mechanistic pathway than PM_2.5_, and while controlling for PM_2.5_ in pollutant mixtures, NO_2_ is found to have associations with allergic diseases, acute respiratory disease exacerbations, cancers and related cellular damage, and stroke [23, 24]. UFP is considered to have a greater toxicity than particles in larger size categories due to its physical characteristics, including a size that allows penetration into the alveoli of the lungs and transport to the bloodstream [25]. While growing evidence implicates the ultrafine PM fraction in a range of adverse health effects including stroke, brain cancer, and childhood respiratory illness, accurately estimating UFP exposure remains a barrier in distinguishing its effects from total PM_2.5_ [25–29].

For NO_2_ and UFP, linear regression-based modeling (land use regression or LUR) has been used to predict the highly localized variation exhibited by these pollutants within national-scale models [18, 19], providing estimates of how exposure varies within cities across the US [8, 30]. However, there are limitations to LUR that affect its representation of both highly localized peaks (< 100 m) and medium-scale intraurban concentration gradients (< 500 m) within a small subdomain. LUR models must assume generalized relationships between geographic predictors and pollution levels, established using a relatively small number of monitoring sites located across a wide geographic area. These relationships may not be representative within a small subset of the model domain, leading to over- or under-estimation around areas with particular features. Additionally, LUR is designed to predict more accurately around the central tendency rather than capturing outliers, and typically does not consider interactions among predictors nor the possibility of other complex nonlinear relationships between predictors and exposure. Thus, near-source peaks and decay patterns may not be represented even if the set of geospatial variables includes close proximity (<50 m) to major roads and point sources. Better understanding how gradients around known local sources differ between empirical and modeled data could inform efforts to adjust or supplement exposure metrics for pollutants known to vary significantly over short distances.

Inaccurate representation of near-source gradients may disproportionately affect exposure estimates for people of color (POC). Observations from previous work evaluating racial/ethnic inequity in air pollution exposure have established that a disproportionate share of POC live closer to highways and major roads as well as point sources such as industrial operations or restaurants [31–35]. In cities where monitoring is sparse, large-domain modeling estimates may be the only near-term option for assessing local exposure inequity. In such cases, targeted knowledge of how possible exposure misclassification relates to readily observed features of the urban environment may inform the design of studies evaluating within-city differences in air pollution exposure.

This study (1) characterizes the discrepancy between high resolution national-scale regression-based predictions and mobile monitoring measurements of two pollutants with high localized variability (UFP and NO_2_), (2) investigates whether this discrepancy is more pronounced near particular features of the urban environment, and (3) examines whether exposure misclassification by LUR predictions differently affects different racial/ethnic groups. We use Bayesian Additive Regression Trees (BART), a machine learning method designed specifically to model and characterize uncertainty about nonlinearities and higher-order interactions that might describe localized hot spots but are typically absent from national-scale regression models. These findings inform the interpretation of intraurban patterns observed in national-scale exposure modeling and motivate future improvements in modeling techniques or measurement error adjustments.

## Methods

### Data collection and processing

#### Hyperlocal observations from mobile monitoring

Spatially continuous measurements from mobile monitoring, for which vehicle-mounted instruments are operated in motion, have been demonstrated to reveal local gradients absent from both regression predictions and fixed-site monitoring [36, 37], and mobile monitoring in multiple domains has identified hot spots associated with known neighborhood features [10, 11, 38]. Mobile monitoring for this study, as described in previous publications, was conducted throughout the 18-month period from May 2015-December 2017 [10, 37]. Two Google Street View vehicles were equipped with the Aclima Ei measurement and data acquisition platform [39], which provides 1 Hz measurements that include NO_2_ mixing ratio via cavity-attenuated phase-shift spectroscopy (Teledyne, Model T500U), UFP count 2.5 nm to >3 µm using a water-based condensation particle counter (TSI, CPC 3788 WCPC), and GPS location data with nominal 1 m precision. Instruments were installed in a passenger vehicle and ambient air was sampled through an inlet mounted on the roof. Vehicles were driven during weekday daytime hours, covering every road segment within assigned areas. The total sampling domain (93 km^2^) comprised thirteen discontinuous domains in the San Francisco Bay Area and surrounding region (Fig. S1) representing a variety of land uses and neighborhood characteristics. Area assignments were distributed across seasons to provide measurements representative of average annual pollution conditions.

Using vehicle GPS coordinates, each 1 Hz on-road measurement was assigned to a unique 100 m × 100 m (0.01 km^2^) grid cell. Previously established methods were followed to evenly weight each unique visit to the grid cell in time-integrated concentration values [22]. For the set of measurements in each grid cell, time stamp data were examined to isolate individual drive passes at that location and calculate the drive pass mean NO_2_ and UFP measurements. A single time-integrated concentration value was calculated for each grid cell as the median of these drive pass means.

#### Land use regression and integrated empirical geographic regression models

We compare mobile monitoring observations of UFP and NO_2_ with predictions from two highly spatially resolved national-scale models that rely on a multiple linear regression framework. Modeled UFP (expressed as particle number count, PNC) is produced as described by Saha et al. (2021) using conventional Land Use Regression (LUR) techniques, including stepwise forward selection of land use variables [19]. This UFP modeling relied on measurements from 38 urban and rural stationary monitoring sites and intensive measurements in three cities. Modeled NO_2_ (ppb) is produced as described by Kim et al. (2020) using an approach termed Integrated Empirical Geographic (IEG) Regression [18], distinguished from a conventional LUR by its expanded set of geospatial prediction variables and a more complex statistical approach. The NO_2_ model was trained on observations from the United States EPA regulatory monitoring sites (*n* = 292). Despite methodological differences, we refer here to both UFP and NO_2_ geographic linear regression models as LUR. For both LUR models, predictions were generated for residential or populated census blocks, the smallest census areal unit. The present analysis considers predictions at the next largest unit, block group, which provides greater spatial coverage by estimating concentrations in unpopulated census blocks via aggregation with populated blocks [18]. Block group predictions are available for download via the Center for Air, Climate, and Energy Solutions [40]. Regression prediction values were assigned to the mobile monitoring grid based on area-weighted averaging of overlapping block groups.

#### Urban feature and population data

Locations of pollution-relevant local features (known local sources, KLS) were obtained from OpenStreetMap (OSM) using the “osmdata” R package [41]. OSM was chosen because it is freely and readily available in urban areas across the United States. We chose seven specific source types categorized as (1) road traffic (residential, arterial, highway, and on-ramp), (2) select commercial operations (food service and gas stations), and (3) industrial operations, identified as locations where land use is described as “industrial”. Features were selected based on known associations with UFP and NO_2_ or precursors of those pollutants. This selection was not intended to identify features that were missing from LUR input data; KLS types are among the many geospatial predictors considered during variable selection for the LUR models considered here. The objective is instead to investigate how local inaccuracies in LUR predictions may relate to nonlinear dynamics around recognized pollution-related infrastructure.

We calculate road density (km road/km^2^), commercial operation density (# locations/km^2^), and industrial land use share (% area labeled “industrial”) for each grid cell based on features located within 50 m, 150 m, and 300 m of the grid cell edge to capture peaks associated with sources in close proximity as well as diffuse concentrations from more distant sources.

Total population and populations of different census-based racial and ethnic identities were obtained for census block groups from the American Community Survey (ACS) for the year 2016 using the “tidycensus” R package [42]. Racial and ethnic groupings were processed to produce five non-overlapping categories: non-Hispanic Asian; non-Hispanic Black; non-Hispanic Native American, Alaskan or Hawaiian, Pacific Islander, one or more races, or other, collectively categorized as “Other”; non-Hispanic white, and Hispanic/Latino. Census block group population data were reallocated to grid cells by area-weighted averaging.

The full set of data used for this analysis shared a common resolution of 0.01 km^2^ and included for each grid cell (a) mobile monitoring observations of UFP and NO_2_, (b) LUR predictions of UFP and NO_2_ from overlapping census block groups, (c) density for each of the seven KLS types and at three buffer sizes, and (d) demographic information including total population and racial/ethnic composition.

### Statistical approach

To characterize the alignment of LUR predictions with local peaks and neighborhood-scale gradients that may be directly observed, we consider mobile monitoring (MM)-based estimates as the benchmark value and consider LUR performance relative to MM. We consider the limitations of regarding MM as a benchmark in the discussion section. We first describe LUR performance in terms of accuracy within the subdomain and ability to identify localized peaks by directly comparing gridded LUR predictions with MM observations, comparing marginal distributions and examining the linear relationship between predicted and observed values. Subsequent analyses use as the dependent variable the localized difference (LD),

1 $${{{{{{\rm{LD}}}}}}}_{{{{{{\rm{i}}}}}}}={{{{{{\rm{MM}}}}}}}_{{{{{{\rm{i}}}}}}}-{{{{{{\rm{LUR}}}}}}}_{{{{{{\rm{i}}}}}}}$$

where MM_i_ is mobile monitoring observations in grid cell i and LUR_i_ is the national LUR prediction resampled to grid cell i. Thus, a positive LD indicates areas where MM observations are greater than LUR predictions. The range of LD reveals the degree to which LUR predictions diverge from observed values across the domain. The divergence between predicted and observed values is termed “localized difference” rather than “error” because estimates of long-term concentrations based on MM are also subject to substantial uncertainty, and in this work only represent estimates of daytime weekday conditions. Nonetheless, MM provides entirely independent high-resolution estimates and has been shown to detect highly localized pollution peaks [10, 11].

We further probe patterns in LD relating to urban features and local population characteristics using a machine learning technique—Bayesian Additive Regression Trees (BART)—with the ability to estimate highly flexible models with nonlinearities and higher-order interactions among variables. Among other uses, this technique has been used in an epidemiological context to investigate relationships between neighborhood-level risk factors and rates of disease [43]. BART, implemented using the bartMachine R package [44], is formulated as a “sum of trees,” where multiple simple regression trees are summed together to produce a larger more complicated regression tree structure for the specified outcome. BART offers several advantages over generalized linear models as it better accommodates prediction of extreme values and potential non-linear relationships among predictors and outcomes. We prioritized these in our modeling efforts for LD specifically because these features are (a) expected to hold relevance at an intraurban scale and (b) very difficult to specify within a national multiple linear regression model. A distinguishing feature of BART relative to other similar tree-based machine learning methods is the full specification of a statistical model so that inferences from BART are based on the posterior distribution of predicted values based on Markov chain Monte Carlo, producing a full account of estimation uncertainty.

#### Modeling localized difference as a function of known local sources

Using BART, we examine whether LD shows significant and potentially non-linear relationships with KLS and whether these relationships may account for local extremes that appear in MM observations but not LUR predictions. We construct a model

2 $${{{{{\rm{LD}}}}}}={{{{{\rm{f}}}}}}({{{{{{\rm{KLS}}}}}}}_{1}\_50,{{{{{{\rm{KLS}}}}}}}_{1}\_150,\ldots ,{{{{{{\rm{KLS}}}}}}}_{7}\_300)+\varepsilon$$

where LD is predicted as a function of each of seven KLS variables at each of three buffer distances (a set of 21 total KLS predictors) and *ε* is a random error with normal distribution centered at zero and variance σ^2^. We consider the overall model fit (pseudo-R^2^ and NRMSE [absolute value of root mean squared error normalized to the observed mean]) to evaluate the share of variability in LD explained by the full set of KLS, and examine the relationship between individual KLS variables and LD using Partial Dependence Plots (PDPs) [45]. From Friedman (2001), the PDP of KLS_j_ gives the average value of predicted LD ($\widehat{LD}$) when KLS_j_ is fixed and the set of all other KLS values varies over their marginal distributions, dP(KLS_-j_), and is estimated by computing

3 $$\widehat{L{D}_{j}}({{{{{\rm{KLS}}}}}}_{j})=\frac{1}{n}\mathop{\sum }\limits_{i=1}^{n}\widehat{LD}({{{{{\rm{KLS}}}}}}_{j},\,{{{{{\rm{KLS}}}}}}_{-j,i})$$

where n is the number of observations and $\widehat{LD}$ denotes predictions via the BART model [44]. The quantity LD_j_(KLS_j_) and its associated posterior uncertainty is then plotted over a range of values for KLS_j_, depicting how $\widehat{LD}$ (*y-*axis, in units of the pollutant modeled) varies as a function of a given KLS-buffer pair (*x*-axis, shown as quantile values of feature density). The PDP includes 95% posterior credible intervals around $\widehat{LD}$. To determine which KLS-buffer pairs provide the most explanatory power for the LD of each pollutant, we calculate the variablinclusion proportion (VIP) for each term and focus our analysis on the eight highest-ranked variables [46].

#### Neighborhood demographics and localized difference

To investigate how LD may differentially accrue among subpopulations, we compare LD across racial/ethnic groups. First, taking an aggregate population perspective, we examine whether the population-weighted distribution of LD differs among groups. Second, taking an area-focused perspective, we examine whether LD varies with the racial/ethnic composition of local residents. Using BART we construct Model A,

4 $${{{{{\rm{LD}}}}}}={{{{{\rm{f}}}}}}({{{{{\rm{Pop}}}}}},\,{{{{{\rm{RE}}}}}}\_{{{{{{\rm{share}}}}}}}_{1},\ldots ,{{{{{\rm{RE}}}}}}\_{{{{{{\rm{share}}}}}}}_{5})+\varepsilon$$

in which LD is predicted by the racial/ethnic composition of the census block groups overlapping each grid cell, where RE_share is the share of population identifying as each of the five racial/ethnic categories, the model controls for total population (Pop), and *ε* is defined as previously. A PDP depicting a positive relationship would indicate that within this local domain, predictions from a national LUR systematically underestimate concentrations in areas with more residents of a particular racial/ethnic group.

To further investigate the relationship between local emission-related infrastructure and differential exposure misclassification by race/ethnicity, we construct Model B,

5 $${{{{{\rm{LD}}}}}} \sim {{{{{\rm{f}}}}}}({{{{{\rm{Pop}}}}}},\,{{{{{\rm{RE}}}}}}\_{{{{{{\rm{share}}}}}}}_{1},\,\ldots ,\,{{{{{\rm{RE}}}}}}\_{{{{{{\rm{share}}}}}}}_{5},{{{{{{\rm{KLS}}}}}}}_{1}\_50,{{{{{{\rm{KLS}}}}}}}_{1}\_150,\,\ldots ,{{{{{{\rm{KLS}}}}}}}_{7}\_300)$$

which considers LD as a function of KLS variables in addition to grid cell population and racial/ethnic composition. We examine the change in race/ethnicity PDPs from Model A to Model B: a weakening of a positive relationship would suggest that the systematic overestimation associated with a specific race/ethnicity can be explained by a higher density of known local sources in neighborhoods with a higher proportion of residents of that race/ethnicity.

## Results

### Characterizing localized difference for the San Francisco Bay Area

**Fig. 1:**
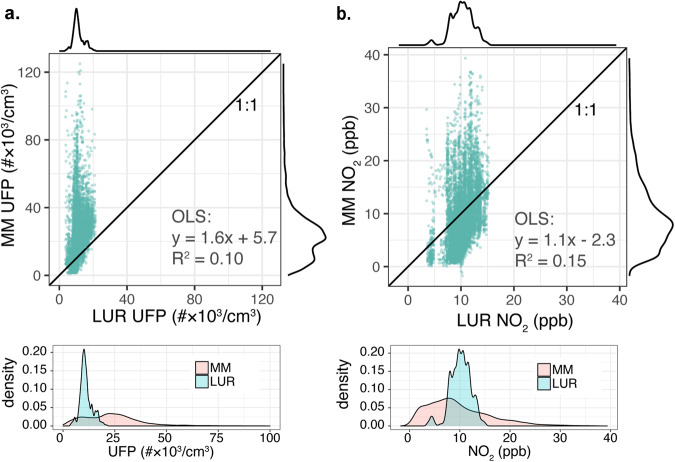
Comparison of LUR predictions with MM observations for each grid cell. These scatter plots show national LUR-based predictions (LUR) versus mobile monitoring observations (MM) for (**a**) UFP, and (**b**) NO_2_, annotated with the linear fit as determined by OLS linear regression. Numerical distributions (kernel density plot) of both datasets are shown in the margins of each figure and overlaid below.

Grid-scaled land use regression predictions (LUR) are directly compared with mobile monitoring observations (MM) in Fig. 1 for UFP (left) and NO_2_ (right). The striated pattern results from variation among grid cells overlapping each census block group, reflecting localized pollution peaks. From OLS regression we find that LUR predictions show a statistically significant linear relationship with MM estimates for both pollutants (*p* < 0.001), but LUR only captures a small proportion of variation among MM values (R^2^ = 0.15 for NO_2_ and 0.10 for UFP). Comparing numerical distributions of LUR and MM values (Fig. 1 inset) further shows the limits of LUR to predict extreme values, as MM features a much wider interquartile range and many values outside the range of LUR predictions. In central tendency, LUR predictions substantially underestimate MM estimates of UFP (medians of 11 and 23 #×10^3^/cm^3^, respectively) but for NO_2_, MM and LUR show similar median values (8.5 vs. 10.2 ppb).

An examination of LD reinforces these findings. The distribution of LD values shows that LUR predictions substantially underestimate UFP and slightly overestimate NO_2_ (Fig. S2). The long upper tail of the LD distribution for both pollutants shows extremes observed via mobile monitoring are not depicted in LUR estimates. Additionally, high LD exists across most LUR values, indicating that peaks occur in areas with both high and low predictions. Geographically, LD encompasses divergence between LUR and mobile observations on multiple spatial scales (Figs. S3–4), with substantial within-neighborhood variation. Neighborhood maps show that the range of LD within most neighborhoods is comparable to the range across the domain, and clusters of high LD appear along roadways—most distinctly for NO_2_—and in locations that may correspond to industrial or commercial land use.

### Relationship between localized difference and known local sources

#### Overall model performance

**Fig. 2:**
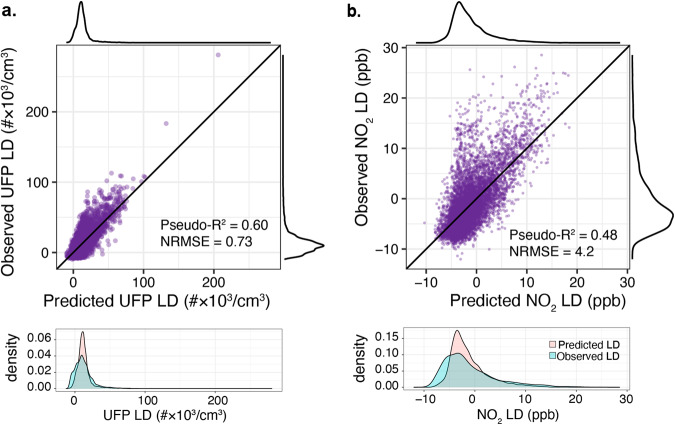
Performance of BART-based model predictions of LD. Predicted localized difference based on BART known local sources model (Eq. 2) compared with observed localized difference training data for UFP (**a**) and NO_2_ (**b**). Numerical distributions (kernel density plot) of both datasets show that predicted LD replicates the observed long upper tail.

We find that using urban feature data, BART-based models successfully reproduce LD patterns, including extreme values. The model of UFP LD as a function of the suite of KLS variables (Eq. 2) predicts the majority of variation (pseudo-R^2^ = 0.60, NRMSE = 0.73) and closely predicts the mean (12.6 versus 12.4 #×10^3^/cm^3^ observed). Importantly, the distribution of predicted LD $(\widehat{LD})$ (Fig. 2) includes a long upper tail, showing that within this framework KLS variables can predict concentration extremes. For NO_2_, the BART fit had pseudo-R^2^ = 0.48 and NRMSE = 4.2 and the $\widehat{LD}$ distribution also includes a long upper tail. Mean NO_2_ $\widehat{LD}$ is −1.2ppb compared with an observed value of -0.9 ppb.

#### Localized difference predicted by individual features

**Fig. 3:**
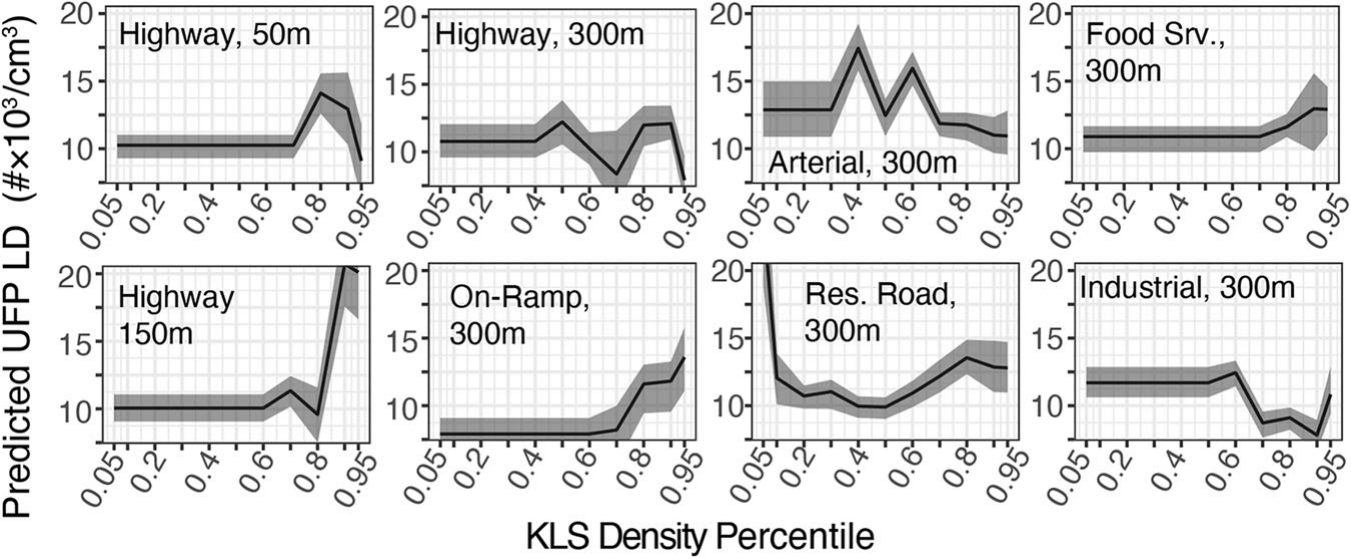
UFP partial dependence plots for select known local sources. Partial dependence of predicted UFP LD on eight categories of known local pollution sources within listed buffer distances.

**Fig. 4:**
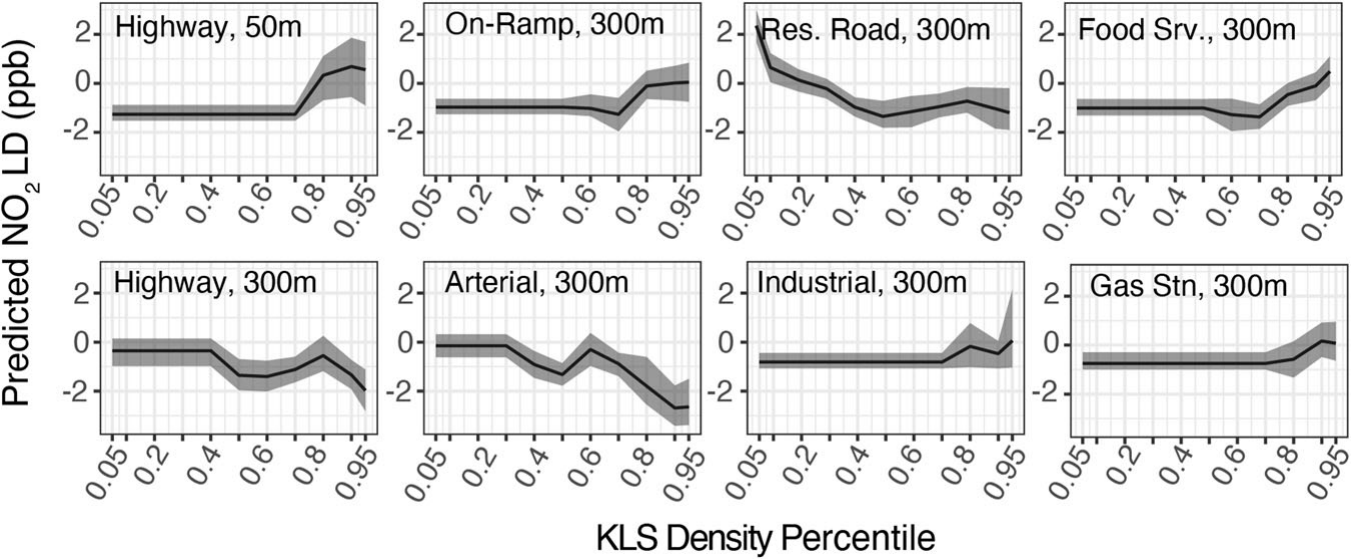
NO2 partial dependence plots for select known local sources. Partial dependence of predicted NO_2_ LD on eight categories of known local pollution sources within listed buffer distances.

Partial dependence plots (PDP) for high variable importance KLS-buffer pairs are shown for UFP (Fig. 3) and NO_2_ (Fig. 4). Many KLS are spatially sparse, with a density of zero over > 50% of the spatial domain. This sparsity produces a presence-absence pattern PDP: a constant $\widehat{LD}$ value across the majority of the domain followed by a slope or stepwise change at the upper end of the distribution. For these features, the key PDP characteristic is the step-change in $\widehat{LD}$ between areas where that feature is absent versus present. KLS with a broader distribution over the domain show more complex partial dependence functions, which may take the form of potentially non-linear positive or negative trends or functions that vary in direction. For these, PDPs may be interpreted in terms of the approximate trend or the amplitude of change for highly nonlinear forms.

For UFP (Fig. 3), the scale of mean $\widehat{LD}$ across plots (varying from 7.5 to 20 #×10^3^/cm^3^) shows that urban feature variables can explain a substantial share of LUR underprediction. Among variables with presence-absence form plots (highways, on-ramps, food service, and industrial land use), most show greater underprediction by national LUR (higher mean $\widehat{LD}$) where present, indicating that peaks observed near these sources via mobile monitoring are underrepresented in nationally modeled estimates of UFP. The greatest magnitude increases in mean $\widehat{LD}$ are associated with the presence of highways within 150 m and 50 m. Lower magnitude increases are associated with the presence of food service and on-ramps within 300 m, and the wider credible interval for locations within 300 m of food service indicates greater model uncertainty predicting $\widehat{LD}$ based on that feature type. An exception to the presence-increase pattern occurs for areas within 300 m of industrial land use: mean $\widehat{LD}$ is predicted to be lower in those areas, although still non-negative. High mean $\widehat{LD}$ in areas with the lowest density of residential roads within 300 m—representing areas with little to no residential population—may reflect the dependence of national-scale LUR predictions on population density as a covariate. Larger buffer sizes tended to have higher VIP (with the exception of highways), suggesting that feature density at moderate spatial scale is more informative for predicting LD.

For NO_2_ (Fig. 4), as with UFP, several spatially sparse feature types show a significant association with LUR underprediction (higher mean $\widehat{LD}$ where present): on-ramps, food service, and gas stations within 300 m and highways within 50 m. However, unlike for UFP, the density of several road types within 300 m were associated with mean $\widehat{LD}$ moving farther below zero, indicating that the national LUR overpredicts in areas with a greater density of residential and arterial roads, and in areas between 50 and 300 m from highways.

### LD and KLS in the context of the racial/ethnic composition of local population

Within this domain, LD does not vary equally across members of different racial/ethnic groups (Fig. S5). Differences are more notable for UFP, with higher medians and more compressed interquartile ranges when weighting by the number of Black or Hispanic/Latino residents, indicating the national LUR systematically underpredicts exposure for these groups. For NO_2_, median LD is similar across groups but IQR bounds differ.

**Fig. 5:**
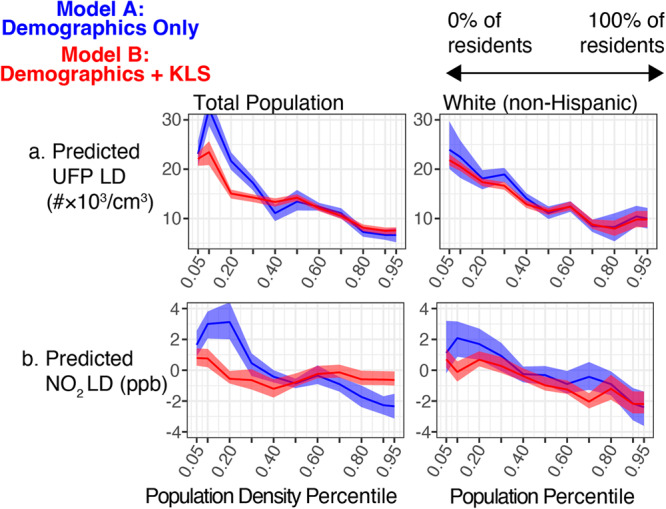
Predicted relationship between LD and population characteristics. Partial dependence of predicted (**a**) UFP LD and (**b**) NO_2_ LD on population density and the share of residents identifying as white non-Hispanic. The blue line indicates LD predicted only using demographic variables (Model A, Eq. 4) and the red line indicates LD predicted based on demographic variables and known local sources (Model B, Eq. 5).

Examining LD modeled as a function of population density and racial/ethnic composition (Model A; Eq. 4), we find that total population and the share of white non-Hispanic residents both show a significant relationship with $\widehat{LD}$ (Fig. 5, in blue), and these models can account for the majority of variation in LD (pseudo-R^2^ = 0.59 for NO_2_, 0.71 for UFP). For both pollutants we see the strongest partial dependence between $\widehat{LD}$ and population, with significantly higher mean $\widehat{LD}$ in low-population areas. Two factors contribute to this relationship: (1) highly localized emissions-intensive activity is more common in non-residential or low-population census blocks, so limitations to LUR representation of local extremes is more acute in areas with low population density; and (2) the national LUR focus on census-based areal units (which are smaller in more densely populated areas) and use of population-weighted averaging for spatial aggregation is designed to maximize accuracy in areas with higher population.

Of particular relevance to environmental justice, there is strong negative partial dependence between $\widehat{LD}$ and the share of white non-Hispanic residents: LD is closer to zero in areas with predominantly white residents, resulting in systematic underestimation of neighborhood-to-neighborhood racial/ethnic exposure inequity. For both pollutants the magnitude of change between the 5th and 95th percentile for share of white residents surpasses that of the partial dependence plots of every KLS previously discussed (5 ppb for NO_2_ and 16 #×10^3^/cm^3^ for UFP), indicating that LD is more strongly related to racial/ethnic composition than the density of any single KLS. Partial dependence relationships do not show clear increasing or decreasing trends for other racial/ethnic groups (Fig. S6), suggesting that underestimation of exposure by national LUR predictions is not specific to one POC identity. This complements the observation from aggregate statistics that the LD distribution for POC populations differs from that of the white non-Hispanic population but are not simply shifted higher or lower, and instead exhibit higher LD in different parts of the numerical distribution (medians and upper/lower quartiles).

To determine whether the relationship between $\widehat{LD}$ and population characteristics is explained by the density of pollution-related infrastructure in lower population or more predominantly POC neighborhoods we look for differences in the slope of population-related PDPs between Model A, described above, and Model B (shown in red in Fig. 5), which includes both demographic and KLS variables as predictors. We find that the addition of pollution source density covariates attenuates the relationship between $\widehat{LD}$ and population across all areas for NO_2_ and in low-population areas (population density below the 30th percentile) for UFP, showing that the greater degree of underestimation by national LUR predictions in low population areas is explained in part by the mischaracterization of pollution gradients near pollution-related infrastructure. The negative trend in $\widehat{LD}$ associated with the share of white non-Hispanic residents, however, is essentially unchanged from Model A to Model B for both pollutants, indicating that disproportionate underprediction of concentrations in areas with majority POC residents cannot be accounted for by factors relating to the considered set of infrastructure covariates.

## Discussion

Consistent with previous studies of the SF Bay Area [37, 47], we find that national LUR models are limited in their ability to characterize air pollution concentrations observed via mobile monitoring, both in their prediction of background concentrations and of localized peaks. We find that mean mobile monitoring estimates of UFP were > 2× higher than LUR predictions while mean NO_2_ was only 10% lower, and intraurban concentration extremes were not represented for either pollutant.

In many contexts, including epidemiology and environmental justice studies, it is more important to determine relative differences in concentration across the urban landscape or among population groups. For example, a pollution-related health effect may be investigated as a function of the distributions of exposure across study members with or without an adverse health outcome, with the detection of effects dependent on differences in exposure distributions between outcome groups and not the absolute magnitude of exposure across the study population. Metrics used to describe environmental injustice often express relative differences in population-weighted average exposure among subgroups or compare exposure distributions among groups (e.g., Atkinson index or subgroup inequity index) [48–50]. For such purposes it is particularly relevant that disagreement between LUR predictions and mobile monitoring estimates is not described by a constant shift in distribution across the geographic domain, nor distributed randomly. Instead, patterns of disagreement evidently relate to features of the urban built environment and population. We find a negative association between LUR underestimation and population: LUR predictions are more representative in areas with high population density. This is not unexpected for the specific LUR predictions examined here, as their modeling prioritized population-scale exposure estimates and was not designed to provide predictions in unpopulated areas [18, 19]. Such optimization is advantageous in evaluating total-population exposure on a large scale. However, this work indicates potential problems when using LUR predictions to evaluate exposure among subpopulations. Additionally, the relationship between LD and readily observed local emission sources suggests that differential distribution of specific populations of interest around those sources may result in exposure differences not fully represented by LUR predictions. Because various types of pollution-related infrastructure predict different patterns of LUR inaccuracy for UFP versus NO_2_, underestimation of exposure inequality due to differential source proximity may vary among pollutants.

An important dimension of the geographic distribution of LD is the misestimation of exposure inequality among racial/ethnic groups. In fact, we find a dependence between the degree of LD and the racial/ethnic composition of local populations: underprediction by national LUR models is higher in areas with a lower share of non-Hispanic white residents. Consequently, exposure misclassification from national LUR predictions is more pronounced for people of color in this spatial domain. The weak representation of highly localized pollution patterns in LUR may contribute to this underprediction: previous work has shown that for PM_2.5_ and traffic-related air pollution, coarser spatial resolution results in an underestimation of average exposures and differences among racial and ethnic groups [6, 51]. However, an examination of local exposure patterns in this domain found that racial/ethnic disparity was most strongly affected by neighborhood-to-neighborhood differences and not hyperlocal peaks [37].

Another factor contributing to differential underprediction may be the lower availability of ground-based measurements to validate model predictions in urban areas with lower residential population and a high share of residents of color [52]. There is a greater overall scarcity of ground-based measurements for UFP than for NO_2_ (38 sites nationwide vs. 272), as unlike NO_2_, UFP is not included in current US EPA National Ambient Air Quality Standards and not routinely measured at regulatory monitoring sites [19]. The lack of representative measurements may contribute to the greater magnitude of geographic variation in UFP $\widehat{LD}$.

This analysis specifically examines a third contributing factor: the influence of localized sources that are not well accounted for in national models [53]. The co-location of local pollution sources with communities of color is a legacy of the dual forces of historically restricted access to housing for people of color in low-pollution neighborhoods and the siting of new pollution sources in areas where residents have less economic and political capital, often communities with a higher share of people of color [32]. We find that the relationship between LD and racial/ethnic composition remained when accounting for proximity to certain urban features shown in other work to be related to higher exposure among people of color, including highways and restaurants [33]. This does not suggest that urban features such as highways and restaurants do not contribute to exposure inequity. It instead suggests that if they do, the resulting inequity may already be accounted for by LUR predictions. However, the persistent patterns in LD across predominantly POC neighborhoods suggest additional unknown factors leading to higher concentrations among these populations that are not captured by the LUR models.

We offer several recommendations based on these findings. First, conclusions drawn about relative exposure differences within a single city based on national LUR predictions should be interpreted with caution. Second, more flexible, non-linear modeling frameworks have the potential to better characterize intraurban concentration gradients and extremes. Third, the limited set of KLS examined here provided substantial predictive power in explaining variation in LD, indicating the usefulness of these factors for downstream measurement error adjustments. Nevertheless, a portion of within-urban variation—including patterns affecting estimates of racial/ethnic exposure disparity—is not explained by the limited set of urban features examined here. The use of a more detailed set of predictors, such as in recent work examining “microscale variables” (e.g., Google Street View imagery and points of interest), may improve modeling of intraurban patterns [47], but the accuracy of within-city exposure disparity estimates would be further improved by integrating community-level knowledge and/or community monitoring [54].

An important limitation to these conclusions comes from the treatment of mobile monitoring observations as the true representation of local pollution patterns. These high-resolution estimates of long-term pollution conditions carry a high degree of uncertainty due to the temporal sparsity of mobile measurements, and some aspects of the sampling design may introduce bias. Mobile monitoring is constrained to on-road sampling, so measurements are made in direct proximity to residential traffic emissions which could inflate MM-based estimates. However, comparison with near-road fixed sites showed that measurements made on residential roads were not systematically higher [36]. In addition, sampling was limited to daytime, weekday hours. Certain types of pollution-generating activity, including traffic and commercial operations, are higher during these hours, so MM is likely to capture more dramatic near-source peaks than are typical over more broadly averaged conditions. These conclusions are thus more accurately interpreted as characterizing the ability of LUR predictions to capture daytime weekday patterns and may be more relevant to segments of the population that tend to spend those hours at home (e.g., young children, older adults, and resident caregivers) than those who do not (e.g., adults working outside of the home). While we do not examine time-activity patterns here, previous work has found exposure estimates for more vulnerable and less advantaged population groups to be least sensitive to the inclusion of time spent traveling and in non-residential locations [55].

Additionally, while these conclusions point to the general usefulness of KLS data, specific conclusions about LD-KLS relationships and racial/ethnic exposure inequity within this relatively restricted geographic domain may not be generalized across other US cities or geographic subdomains. Partial dependence plots also do not represent direct relationships between KLS emissions and pollution: confounding by other neighborhood features is likely, and in some cases offers a plausible explanation for observed relationships (e.g., food service is not a dominant source of urban NO_2_, so dependence between the two may be explained by vehicle traffic or other activity geographically associated with food service).

Publicly available, census block-scale LUR-based air pollution predictions provide useful insight into national-scale patterns of air pollution exposure. While these models are not designed to replicate local patterns within individual cities, predictions might be used in lieu of local monitoring due to resource and time constraints. It is important to understand how studies that utilize these predictions within a restricted geographic area may be affected by systematic prediction errors, especially for pollutants with strong near-source peaks and high intraurban variability like NO_2_ and UFP. Comparing national predictions with highly spatially resolved local observations, we have documented relationships between the divergence of predicted and observed values with both urban features associated with local emission peaks and with population characteristics. The first set of relationships indicate the potential of using known local sources to resolve exposure misclassification, while the second set reveals that national population-weighted models pose a risk of systematically mischaracterizing exposures for some racial/ethnic subpopulations. These findings can inform future studies investigating intraurban exposure patterns using national LUR estimates and motivate further improvements in modeling for local domains.

## Supplementary information

Supplementary Information

## Data Availability

Code in R for data processing, graphics generation, and replicating this analysis are available from https://github.com/SEChambliss-AQ/LD-analysis/ and the full set of data to run this code is available from 10.5281/zenodo.10120281.
