# Supplementary material for: Local exposure misclassification in national models: relationships with urban infrastructure and demographics
Source: J Expo Sci Environ Epidemiol. 2023 Dec 22;34(5):761–9. doi: 10.1038/s41370-023-00624-z (PMC11446823; doi:10.1038/s41370-023-00624-z)
Supplement: Supplementary file 1 — Supplementary Information [file 41370_2023_624_MOESM1_ESM.docx]

**TABLES**

**Table S1. Summary statistics for mobile monitoring observations (MM) and land use regression predictions (LUR) within the sampling domain**

|  |  | Mean | Median | IQR | 10th pctl | 90th pctl |
| --- | --- | --- | --- | --- | --- | --- |
| NO_2_ | MM | 9.78 | 8.54 | 8.15 | 2.56 | 18.62 |
|  | LUR | 10.17 | 10.24 | 2.65 | 7.95 | 12.80 |
| UFP | MM | 24.12 | 22.67 | 17.29 | 7.20 | 40.49 |
|  | LUR | 11.08 | 10.53 | 3.16 | 8.02 | 15.65 |

**FIGURES**

**
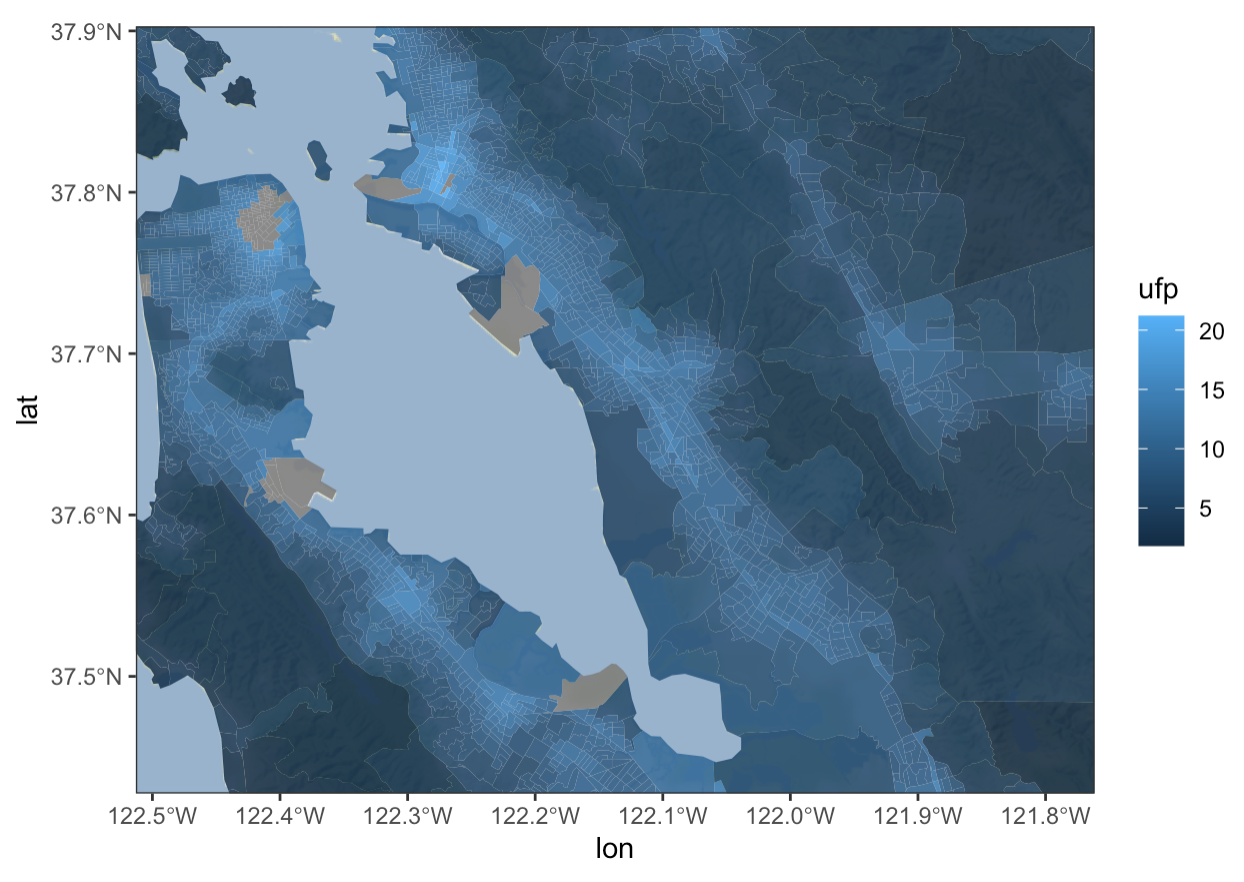

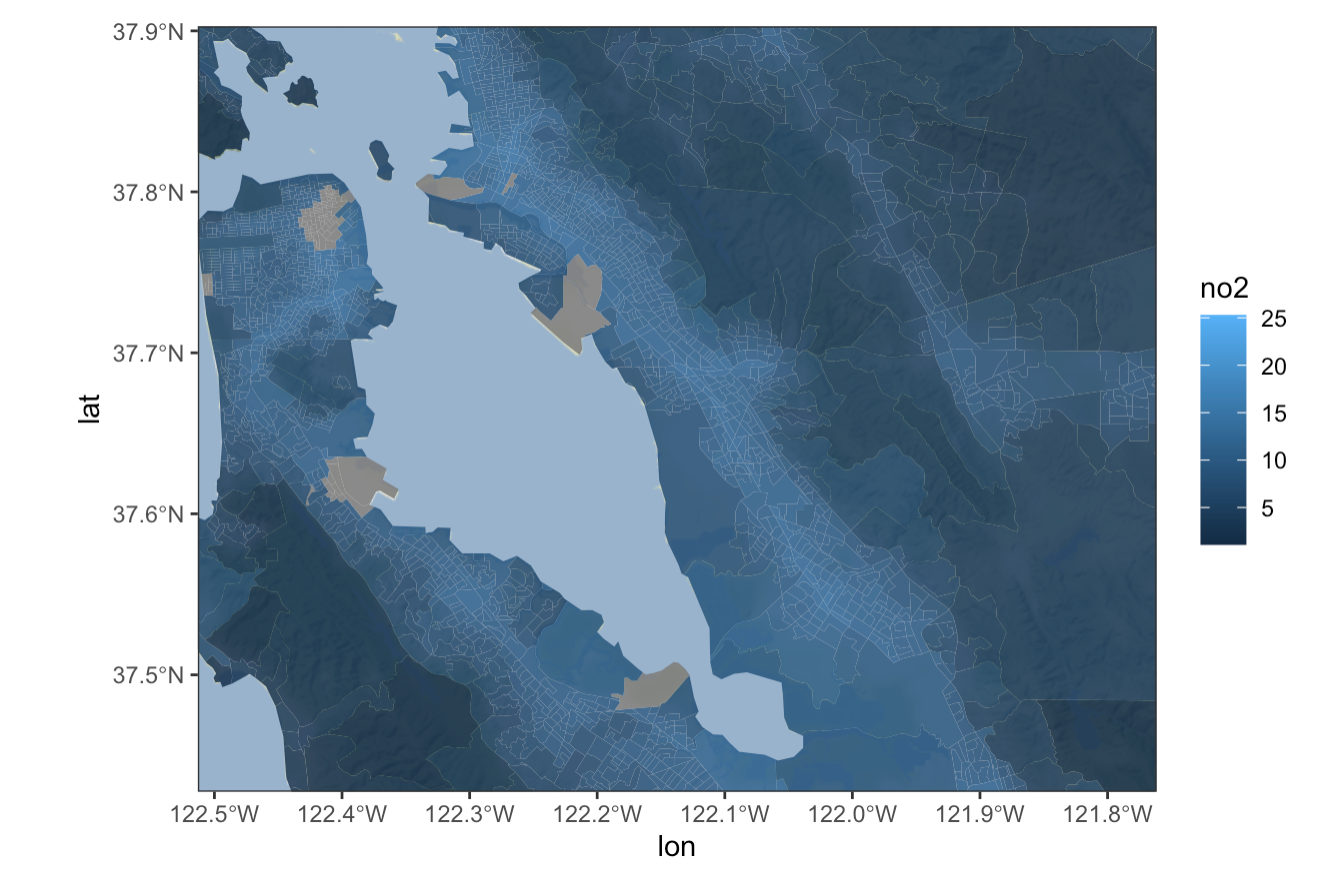
**

**Figure S1.** Maps of LUR predictions of (a) ultrafine particle count (#×10^3^/cm^3^) and (b) NO_2_ mixing ratio (ppb) within the San Francisco Bay Area. Gray areas show census blocks for which predictions are not provided in publicly available data.


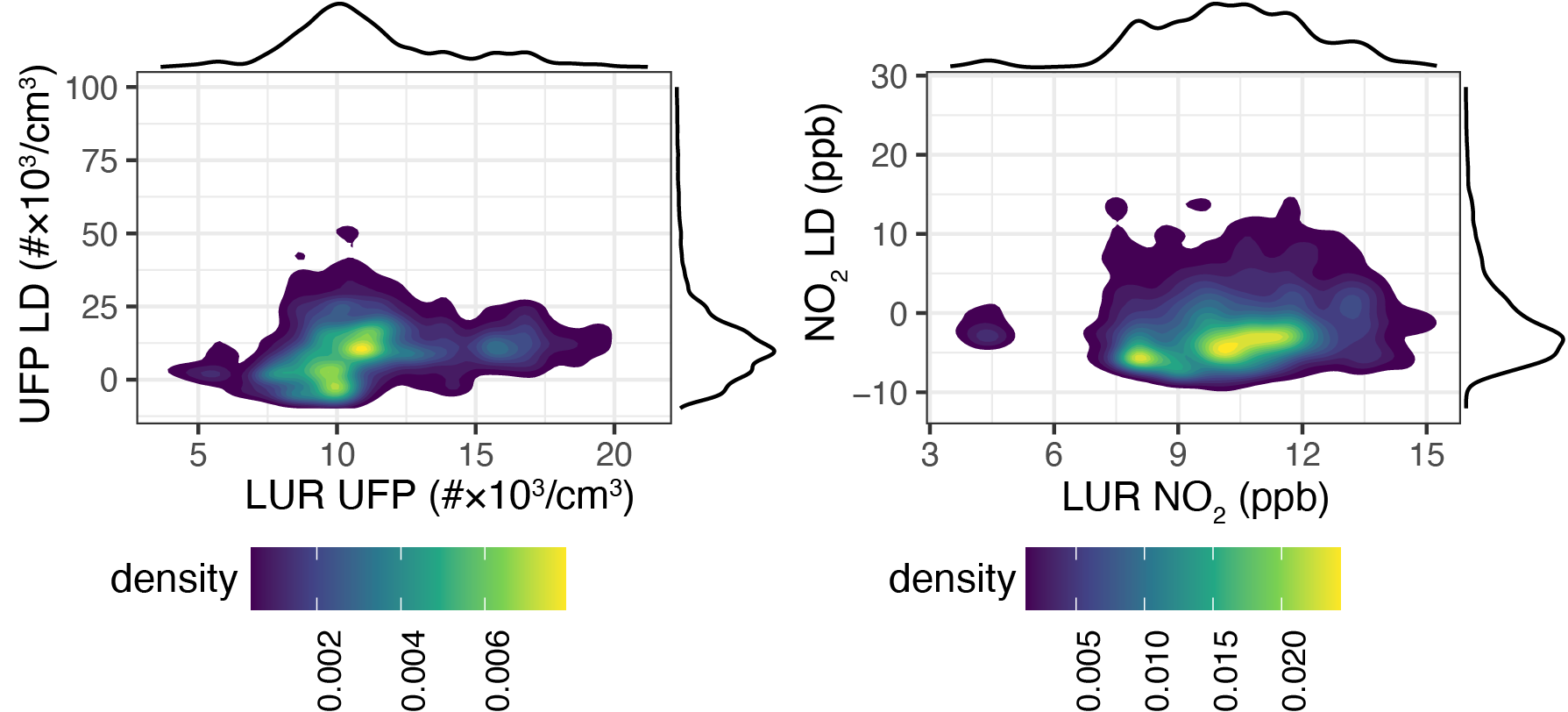


**Figure S2.** Distribution of localized difference as function of LUR.


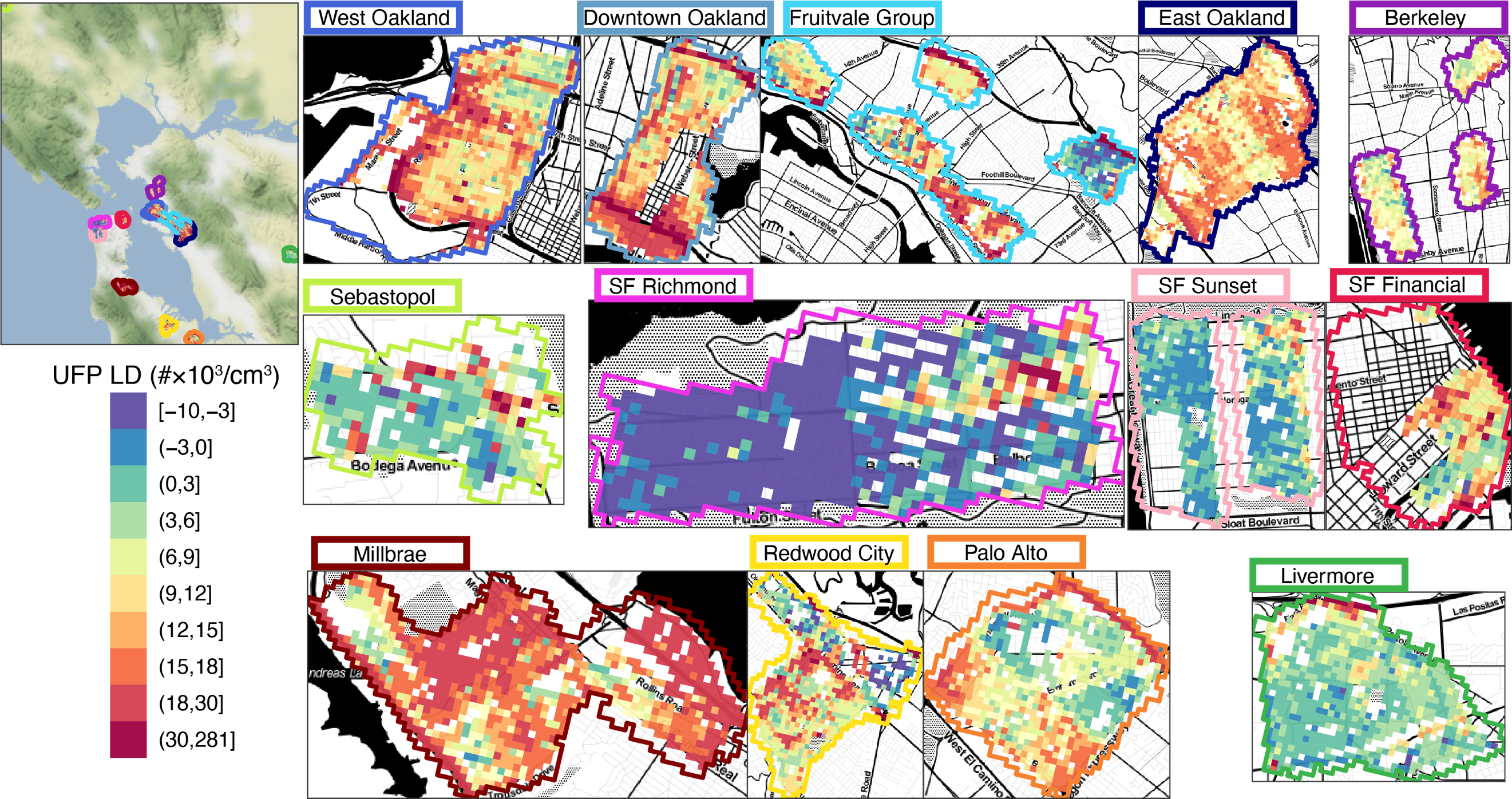


**Figure S3.** Ultrafine particle count (UFP) Localized Difference metric (LD, Eq. 1) at 100 m grid resolution within the thirteen mobile monitoring neighborhoods. Neighborhood locations are shown in the inset map with colors corresponding to neighborhood and label outlines. A positive LD indicates areas where mobile monitoring observations are greater than LUR predictions. These UFP LD maps reveal patterns of divergence between mobile monitoring observations and national LUR predictions at both highly localized scales, appearing as differences between neighboring grid cells, and moderate intraurban scales, appearing as broader clustering patterns within neighborhoods. The range of within-neighborhood variation in UFP LD is comparable to the range across the domain; most neighborhoods include areas where LUR predictions align well with observed concentrations (LD close to zero) and clusters where observed concentrations exceed predictions by >18 #×10^3^/cm^3^ (for reference, median LUR-predicted UFP is 11 #×10^3^/cm^3^). Clusters of high LD appear along arterials and highways (most clearly seen along the edges of Oakland neighborhoods, as well as Palo Alto and Millbrae) as well as around commercial districts (most distinct in the San Francisco neighborhoods of Richmond and the Financial District); these apparent associations are investigated further using BART modeling.


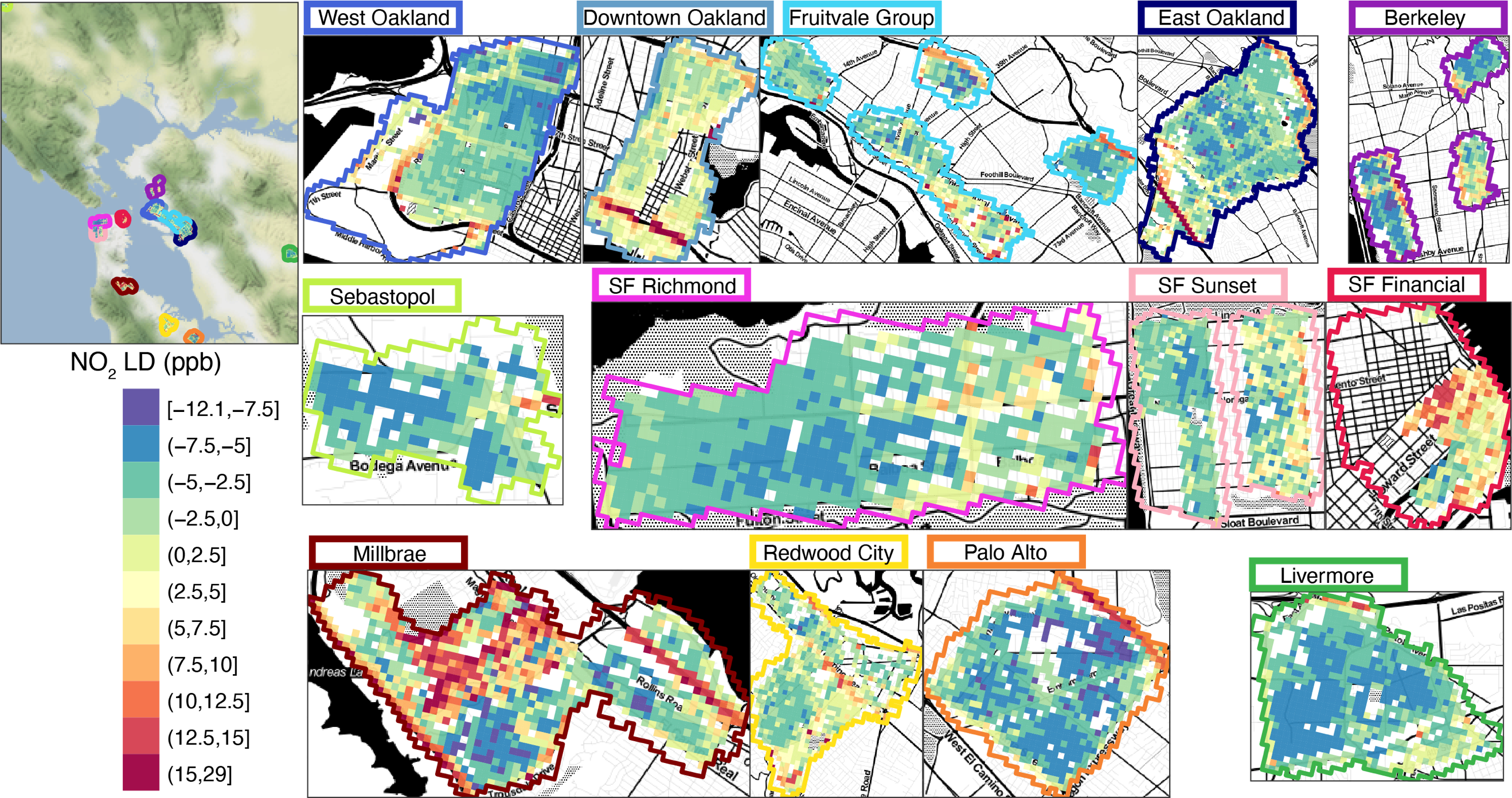


**Figure S4.** NO_2_ Localized Difference metric (LD, Eq. 1) at 100 m grid resolution within the thirteen mobile monitoring neighborhoods. Neighborhood locations are shown in the inset map, with colors corresponding to neighborhood and label outlines. A positive LD indicates areas where mobile monitoring observations are greater than LUR predictions. As with UFP LD maps, these NO_2_ LD maps reveal patterns of divergence between mobile monitoring observations and national LUR predictions at both highly localized scales, and the range of within-neighborhood variation in NO_2_ LD is comparable to the range across the domain. In primarily residential areas of most neighborhoods, LUR predictions tend to exceed observed NO_2_ values resulting in negative LD. The most distinct patterns of higher LD appear along highways and major arterials.

**
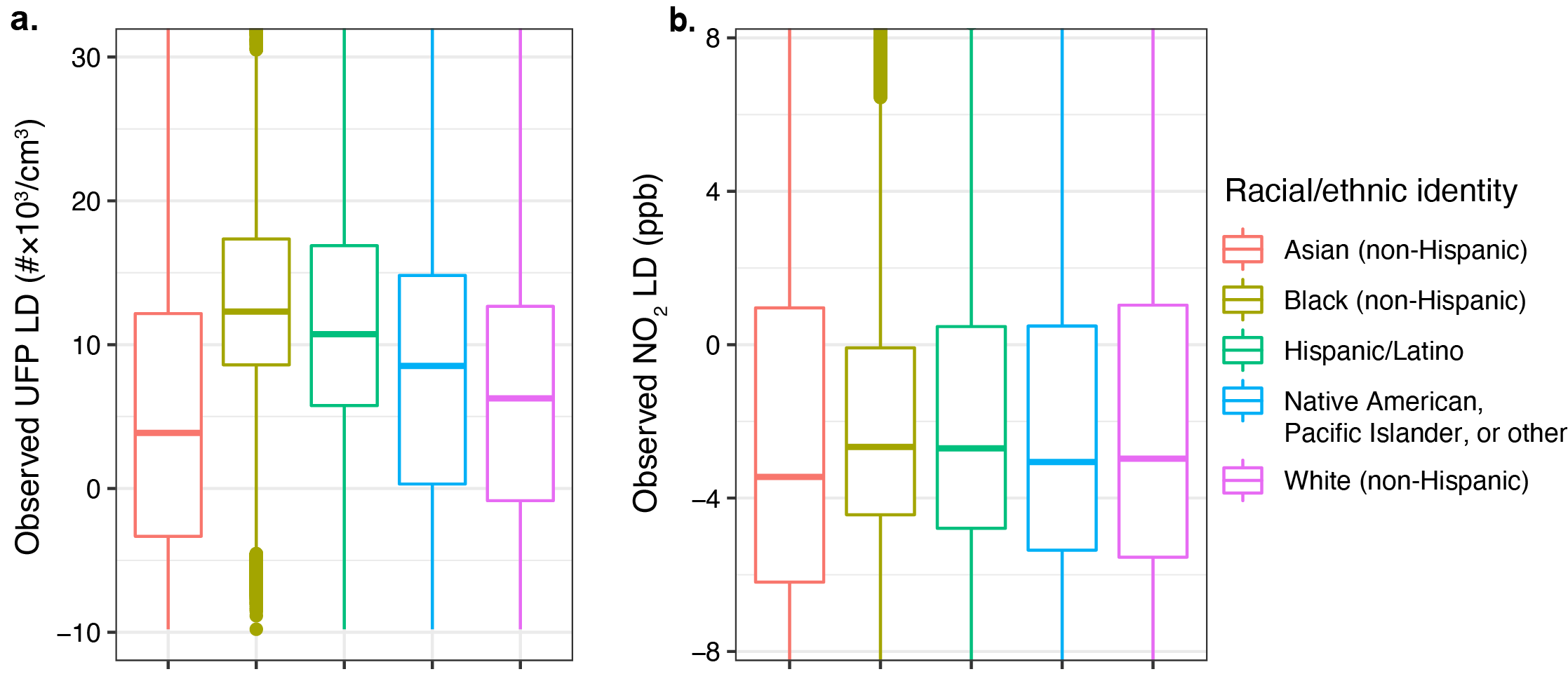
Figure S5.** Population-weighted distributions of LD for (a) UFP and (b) NO_2_ for five census-based racial/ethnic groups.

**
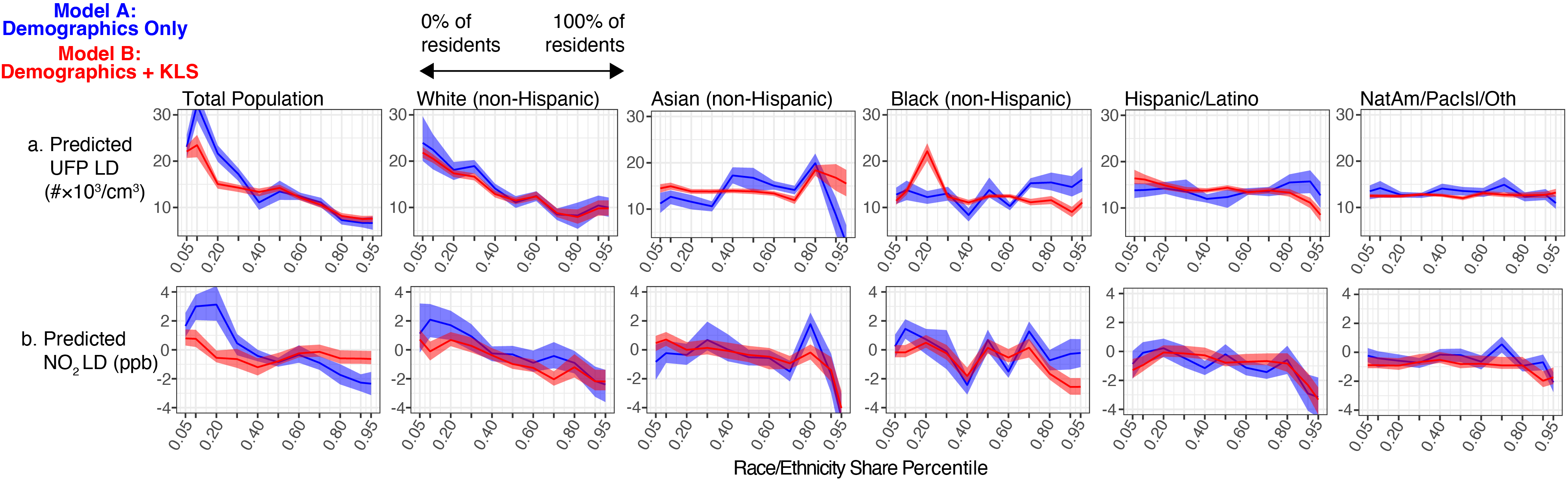
Figure S6.** Partial dependence of predicted (a) UFP LD and (b) NO_2_ LD on population density and the share of residents identifying as each of five racial/ethnic groups. The blue line indicates LD predicted only using demographic variables (Model A, Eq. 4) and the red line indicates LD predicted based on demographic variables and known local sources (Model B, Eq. 5).
